# Red and Processed Meat Intake Is Associated with Higher Gastric Cancer Risk: A Meta-Analysis of Epidemiological Observational Studies

**DOI:** 10.1371/journal.pone.0070955

**Published:** 2013-08-14

**Authors:** Hongcheng Zhu, Xi Yang, Chi Zhang, Chen Zhu, Guangzhou Tao, Lianjun Zhao, Shaowen Tang, Zheng Shu, Jing Cai, Shengbin Dai, Qin Qin, Liping Xu, Hongyan Cheng, Xinchen Sun

**Affiliations:** 1 Department of Radiation Oncology, The First Affiliated Hospital of Nanjing Medical University, Nanjing, China; 2 Department of Epidemiology and Biostatistics and Ministry of Education Key Laboratory for Modern Toxicology, School of Public Health, Nanjing Medical University, Nanjing, China; 3 Department of Radiation Oncology, Huai'an First People's Hospital, Nanjing Medical University, Huai'an, China; 4 The Comprehensive Cancer Center, Nanjing Drum Tower Hospital and Clinical College of Nanjing Medical University, Nanjing, China; 5 Department of Epidemiology and Biostatistics, School of Public Health, Peking University Health Science Centre, Beijing, China; 6 Tumor Institute, Nantong Tumor Hospital, Nantong, China; 7 Department of Oncology, Taizhou People's Hospital, Taizhou, China; 8 Department of Synthetic Internal Medicine, The First Affiliated Hospital of Nanjing Medical University, Nanjing, China; Peking University Cancer Hospital and Institute, China

## Abstract

**Background:**

Red and processed meat was concluded as a limited-suggestive risk factor of gastric cancer by the World Cancer Research Fund. However, recent epidemiological studies have yielded inconclusive results.

**Methods:**

We searched Medline, EMBASE, and the Cochrane Library from their inception to April 2013 for both cohort and case-control studies which assessed the association between red and/or processed meat intake and gastric cancer risk. Study-specific relative risk estimates were polled by random-effect or fixed-effect models.

**Results:**

Twelve cohort and thirty case-control studies were included in the meta-analysis. Significant associations were found between both red (RR: 1.45, 95% CI: 1.22–1.73) and processed (RR: 1.45, 95% CI: 1.26–1.65) meat intake and gastric cancer risk generally. Positive findings were also existed in the items of beef (RR: 1.28, 95% CI: 1.04–1.57), bacon (RR: 1.37, 95% CI: 1.17–1.61), ham (RR: 1.44, 95% CI: 1.00–2.06), and sausage (RR: 1.33, 95% CI: 1.16–1.52). When conducted by study design, the association was significant in case-control studies (RR: 1.63, 95% CI: 1.33–1.99) but not in cohort studies (RR: 1.02, 95% CI: 0.90–1.17) for red meat. Increased relative risks were seen in high-quality, adenocarcinoma, cardia and European-population studies for red meat. And most subgroup analysis confirmed the significant association between processed meat intake and gastric cancer risk.

**Conclusions:**

Our findings indicate that consumption of red and/or processed meat contributes to increased gastric cancer risk. However, further investigation is needed to confirm the association, especially for red meat.

## Introduction

Although the incidence of gastric cancer has decreased steadily over the last 50 years worldwide, the malignancy remains the second leading cause of cancer death globally [1], [2]. Identification of risk factors amenable for modification could play a remarkable role in the morbidity and mortality of the cancer. Infection with *Helicobacter pylori* is an established risk factor for non-cardia gastric cancer; however, only a small proportion of those infected go on to develop gastric cancer [2], suggesting the contribution of other risk factors.

Meat consumption has risen in developed and developing countries and the intake of red and/or processed meat is a potential risk factor of gastric cancer [3]. The endogenous formation of carcinogenic N-nitroso compounds is influenced by the heme content of meat, particularly red meat. N-nitroso compounds (NOCs) are also formed in processed meat containing high amount of salt, nitrate and nitrite compounds [4]. Other carcinogens of heterocyclic amines and polycyclic aromatics hydrocarbons are formed during the cooking of meat at high temperatures [5]. While several studies have found positive association between red and processed meat intake and gastric cancer risk, a comprehensive review by the World Cancer Research Fund concluded that the evidence was “limited-suggestive” due to insufficient data mostly from case-control studies [6].

Since whether there is association between red and/or processed meat intake and gastric cancer risk remains uncertain, we conducted this systematic review and meta-analysis for more sufficient evidence on this issue.

## Methods

### Search strategy

A computerized literature search was conducted in MEDLINE (PubMed, http://www.ncbi.nlm.nih.gov/pubmed/), EMBASE (www.embase.com/), and the Cochrane Library (http://www.thecochranelibrary.com/) from their inception to April 10, 2013, by two independent investigators (Zhu and Yang). We searched relevant studies using the following medical subject heading terms and/or text words: *“gastric cancer”, “gastric neoplasm”, “stomach cancer”, “stomach neoplasm”* in combination with *“meat”, “red meat”, “processed meat”, “preserved meat”, “beef”, “veal”, “pork”, “lamb”, “ham”, “sausage”, “bacon” “hot dogs” and “salami”.* In addition, we carried out a broader search on diet or foods and gastric cancer and check the reference lists of retrieved articles and relevant review articles so as to identify additional relevant studies. No language restrictions were imposed.

### Eligibility criteria

Red and processed meat was defined according to Word Cancer Research Fund/American Institute for Cancer Research in our meta-analysis [6]. Studies were included if these *1)* had a case-control or cohort design; *2)* evaluated the association between red meat and/or processed meat intake and gastric cancer risk; *3)* presented odds ratio (OR), relative risk (RR) or hazard ratio (HR) estimates with 95% confidence interval (CI). If the publications were duplicated or articles from the same study population, the publication with a larger size was included. Non-peer-reviewed articles, ecologic assessments, correlation studies, experimental animal studies and mechanistic studies were excluded.

### Data extraction and quality assessment

Two independent researchers (Zhu and Yang) extracted the following data from each study that met the criteria for inclusion: the first author's name, year of publication, geographic regions, journal, number of cases, cohort size, cohort name and duration of follow-up (cohort studies), number and type of control subjects (case-control studies), type of cancer, type of meat, consumption categories, adjusted ORs, RRs, or HRs with 95%CI, and adjusted variables. When several risk estimates were presented for men and women, each type of gastric cancer, or a single type of meat, the detailed information were extracted.

A 9-star system on the basis of the Newcastle-Ottawa Scale was used to assess the study quality on 3 broad perspectives [7]. Considering that there is possibly a direct or indirect caloric intake and gastric cancer risk, an energy-adjusted residual or nutria-density model was added as an item for modification of the scoring system [8]. Hence, the full scores was 10 stars, and a study with ≥7 awarded stars was defined the high-quality study.

### Statistical analysis

Statistical analysis was based on comparison of the highest intake category with the lowest intake category (which may include people do not eat red or processed meat). The study-specific most adjusted association estimates were used as the common measure of association across studies and the ORs were considered to be equivalent to RRs or HRs because gastric cancer is a rare outcome in humans. If association estimates were provided separately of different sex or subtypes of cancer, combined RRs and CIs were used in overall analysis.

Meta-analysis of total red/processed meat and a single type of red/processed meat (beef/pork/bacon/ham/sausage) were both included. Subgroup analysis of red/processed meat was conducted by study quality, study design (cohort studies and case-control studies), control source (population–based and hospital-based), sex (men and women), histologic subtype (adenocarcinoma), anatomical subtype (cardia and non-cardia), geographic region (Asia, Europe, North America), outcome (incidence), and study adjustments (smoking, alcohol drinking, total energy intake, family history, and body mass index adjustments). Due to the limited number of studies (≤2) that reported risk estimates of mortality and Latin America, some subgroup analysis on these issues was not present in the final table of results.

The possible heterogeneity in results across studies was examined by using the Cochran *Q* and *I^2^* statistics [9]. The null hypothesis that the studies are homogeneous was rejected if the *P* value for heterogeneity was <0.05 or the *I^2^* was ≥50%. When substantial heterogeneity was detected, the summary estimate based on the random effects model was reported [10]. Otherwise, the summary estimate based on the fixed effects model was reported [11].

Publication bias was evaluated by generating funnel plots for a visual examination, conducting correlation and regression tests for significance, and using Egger's linear regression [12] and Begg's rank correlation [13] methods. A *P* value of <0.05 for the two aforementioned tests was considered representative of significant statistical publication bias. All statistical analyses were performed by using STATA (version 11.0; StataCorp, College Station, Texas, USA).

## Results

### Literature search

**Figure 1:**
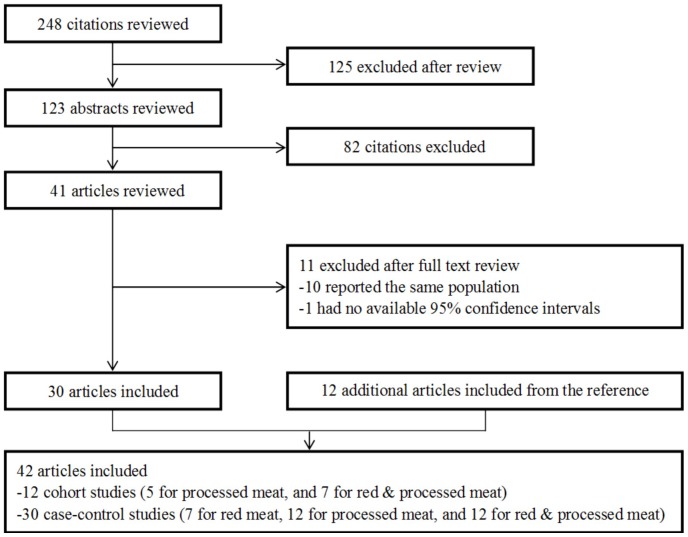
Reference searched and selection of studies in the meta-analysis.

The search strategy generated 248 citations, of which 42 were identified in the final analysis (Figure 1). All of the studies were published form 1985–2012, consisting of 12 [14]–[25] cohort studies (7 for processed meat, and 5 for red & processed meat) and 30 [26]–[55] case-control studies (7 for red meat, 12 for processed meat, and 11 for red & processed meat). Ten [56]–[65] articles were excluded because other articles of the same studies with more cases or with information required in the analysis were already included. One [66] article was excluded due to no 95% confidence intervals presented and there was no original data to calculate it.

### Study characteristics and quality assessment

Characteristics of the included studies are shown in Table S1 and Table S2. The twelve cohort [14]–[25] studies were consisted of a total of 2343450 participants and 5118 gastric cancer cases, and the thirty [16]–[55] case-control studies were involved in 11680 cases and 67544 controls. The outcome was in incidence most of the studies, while mortality from gastric cancer was presented in three [15], [18], [19] studies. Eleven [19], [21], [28], [32], [38], [42]–[44], [48], [54], [55] studies were conducted in Asia, sixteen [17], [20], [22], [23], [25], [27], [29], [30], [ 31], [33], [34], [36], [40], [41], [47], [49] in Europe, eleven [14]–[16], [18], [24], [26], [37], [ 39], [45], [50]–[52] in North America, and four [35], [39], [46], [53] in Latin America. Two [14], [15] studies were population of only men and two [23], [44] were only women. All of the studies provide RR or OR for the highest versus the lowest intake, while one [15] just provide a per 100 g increment OR for the association between processed meat and gastric cancer risk and one [52] provide per one serving/day for red meat. One [32] case-control reported ORs using population and hospital controls, so both of the available data was extracted. In most studies, relative risk estimates were adjusted for age and sex. Many were adjusted for education, residence, smoking, drinking, body mass index, total energy and a variety of other nutrients intake. Seventeen [22]–[25], [36]–[38], [40], [41], [43], [46], [47], [50], [51], [53]–[55] studies were involved in the analysis of the association between total red meat intake and gastric cancer and twenty-seven [14], [16]–[19], [22]–[25], [28], [29], [31], [32], [37], [ 39], [41], [42], [44]–[46], [48]–[51], [53], [54] studies were included for total processed meat. Sixteen [15], [20], [21], [23], [27], [30], [31], [33]–[35], [37], [43]–[45], [ 48], [53] studies reported data of a single kind meat (such as beef, pork, ham, sausage, or bacon). Hence, analyses of individual meat items were also conducted.

The quality score of included studies ranged from four to ten stars on the scale, the median score was 7. The median scores of cohort and case-control studies were 8 and 6, respectively. High-quality studies (with a sore more than 6) included ten cohort studies and thirteen case-control studies. The study-specific quality score are summarized in Table S3 and Table S4.

### Red meat and gastric cancer

**Figure 2:**
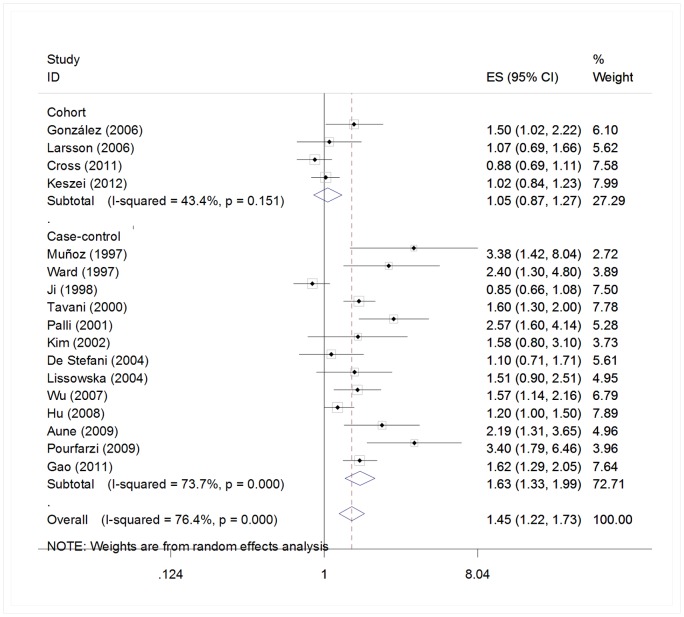
Estimates (95% CIs) of red meat intake (highest versus lowest category) and gastric cancer risk. Squares indicate study-specific relative risks (size of the square reflects the study-specific statistical weight, i.e., the inverse of the variance); horizontal lines indicate 95% confidence intervals; diamond indicates summary relative risk estimate with its corresponding 95% confidence interval.

**Figure 3:**
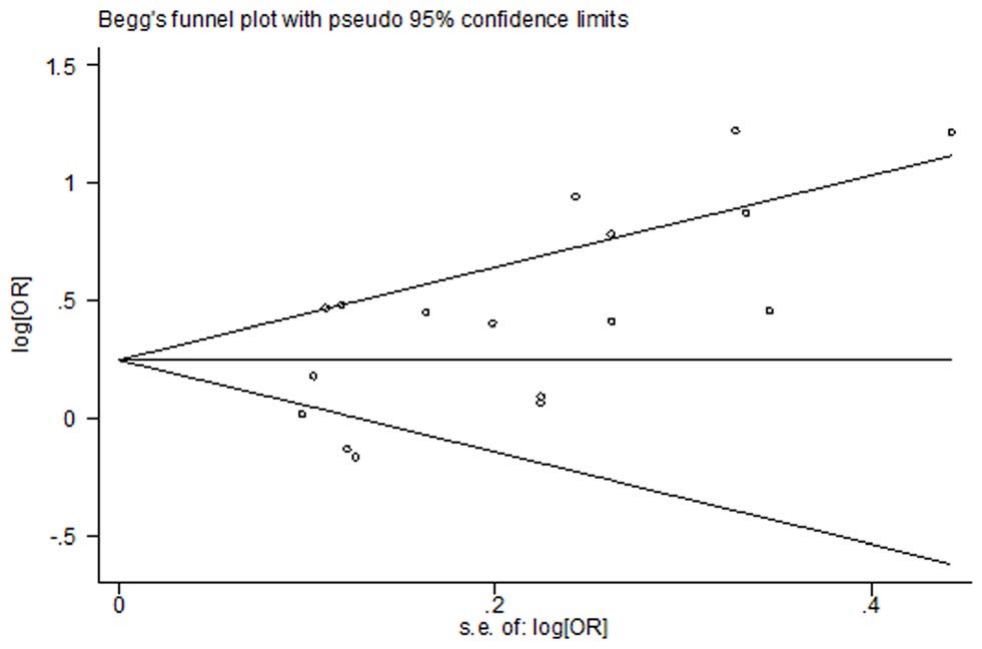
Beeg's test of studies for red meat intake and gastric cancer risk.

Among the five [21]–[25] cohort and seventeen [27], [35]–[38], [ 40], [41], [43], [44], [46]–[48], [50], [51], [53]–[55] case-control studies for red meat, four [22]–[25] cohort and thirteen [36]–[38], [40], [41], [43], [46], [47], [50], [51], [53]–[55] case-control studies are included in the meta-analysis of total red meat intake and gastric cancer risk in the highest versus lowest model, others are excluded because a single kind of red meat [21], [27], [35], [37], [43], [44], [48], [53] or a continues model of data [52] was reported. We found that high intake of red meat is associated with a 45% increased risk of gastric cancer (RR = 1.45, 95% CI = 1.22–1.73). (Figure 2) Statistically significant heterogeneity was detected (*Q* = 67.92, *P*<0.001, *I^2^* = 76.4%), and publication bias was indicated from Egger's test (*P* = 0.015) but not Beeg's test (*P* = 0.118) (Figure 3). In the analysis of individual red meat items, high beef consumption was associated with a 28% increased risk of gastric cancer (RR = 1.28, 95% CI = 1.04–1.57) with no heterogeneity (*Q* = 6.59, *P* = 0.47, *I^2^* = 0%) and publication bias (Egger's test: *P* = 0.849). No significant association was found between pork and gastric cancer risk (RR = 1.31, 95% CI = 0.97–1.78).

In subgroup analysis for red meat, the results were fairly consistent with the overall summary measure when the analysis were restricted to high-quality studies (RR = 1.26, 95% CI = 1.00–1.59), case-control studies (total: RR = 1.63, 95% CI = 1.33–1.99; population controls: RR = 1.64, 95% CI = 1.17–2.28; hospital controls: RR = 1.61, 95% CI = 1.41–1.85), adenocarcinomas (RR = 1.28, 95% CI = 1.06–1.54) and cardia (RR = 1.26, 95% CI = 1.05–1.52). However, no significant association was observed between cohort studies (RR = 1.02, 95% CI = 0.90–1.17), as well as the subgroup of sex (men: RR = 1.06, 95% CI = 0.89–1.26, women: RR = 0.88, 95% CI = 0.71–1.08) and noncardia (RR = 1.26, 95% CI = 0.92–1.71). In subgroup analysis by geographic reign, positive association was found between European populations (RR = 1.52, 95% CI = 1.16–2.00), while null results were found among Asian (RR = 1.56, 95% CI = 0.93–2.63) and North American studies (RR = 1.30, 95% CI = 0.94–1.79). In the adjustments models, positive association was found when studies adjusted for smoking (RR = 1.26, 95% CI = 1.04–1.52), total energy intake (RR = 1.35, 95% CI = 1.08–1.70), family history (RR = 2.46, 95% CI = 1.77–3.44) and BMI (RR = 1.29, 95% CI = 1.04–1.60), and null result was found for alcohol drinking (RR = 1.17, 95% CI = 0.97–1.40). (Table 1).

**Table 1:** Summary relative risks (RRs) of the association between red and processed meat consumption and gastric cancer risk^a^.

	No. of studies	No. of cases	Reference	RR (95%CI)	Test of heterogeneity		
					*Q*	*P*	*I^2^*%
Overall studies							
Red meat	17	8484	22, 23, 24, 25, 36, 37, 38, 40, 41, 43, 46, 47, 50, 51, 53, 54, 55	**1.45 (1.22–1.73)**	67.92	<0.001	76.4
Beef	8	2625	21, 27, 35, 37, 43, 44, 48, 53	**1.28 (1.04–1.57)**	6.59	0.47	0
Pork	5	1968	21, 35, 43, 44, 48	1.31 (0.97–1.78)	6.54	0.162	28.9
Subgroup analyses for red meat							
High-quality studies (score≥7)	9	3766	22, 23, 24, 25, 41, 43, 46, 47,50	**1.30 (1.05–1.61)**	24.79	0.002	67.7
Study design							
Cohort studies	4	2111	22, 23, 24, 25	1.02 (0.90–1.17)	5.30	0.151	43.4
Case-control studies	13	6373	36, 37, 38, 40, 41, 43, 46, 47, 50, 51, 53, 54, 55	**1.63 (1.33–1.99)**	45.69	<0.001	73.7
Population-based controls	7	3974	37, 38, 41, 47, 50, 51, 54	**1.64 (1.17–2.28)**	33.60	<0.001	82.1
Hospital-based controls	6	2399	36, 40, 43, 46, 53, 55	**1.61 (1.41–1.85)**	7.09	0.214	29.5
Sex							
Men	3	1406	25, 38, 46	1.06 (0.89–1.26)	1.17	0.558	0
Women	4	765	23, 25, 38, 46	0.88 (0.71–1.08)	2.48	0.479	0
Histologic type							
Adenocarcinoma	8	5091	22, 24, 25, 37, 46, 50, 51, 55	**1.28 (1.06–1.54)**	24.21	0.001	71.1
Anatomical subtype							
Cardia	5	1567	22, 24, 25, 50, 55	**1.26 (1.05–1.52)**	6.60	0.158	39.4
Non-cardia	5	1831	22, 24, 25, 50, 55	1.26 (0.92–1.71)	15.36	0.004	74.0
Geographic region							
Asia	4	2388	38, 43, 54, 55	1.56 (0.93–2.63)	23.84	<0.001	87.4
Europe	7	2645	22, 23, 25, 36, 40, 41, 47	**1.52 (1.16–2.00)**	23.59	0.001	74.6
North America	4	2936	24, 37, 50, 51	1.30 (0.94–1.79)	13.48	0.004	77.7
Adjustments							
Smoking, yes	9	6178	22, 24, 25, 38, 40, 47, 50, 51, 52	**1.26 (1.04–1.52)**	33.63	<0.001	76.2
Alcohol drinking, yes	8	5785	22, 23, 24, 25, 38, 40, 50, 51	1.17 (0.97–1.40)	26.91	<0.001	74.0
Total energy intake, yes	9	3905	22, 23, 24, 25, 41, 46, 47, 50, 53	**1.35 (1.08–1.70)**	29.65	<0.001	73.0
Family history, yes	3	731	41, 43, 53	**2.46 (1.77–3.44)**	2.65	0.266	24.6
BMI, yes	8	4465	23, 24, 25, 41, 46, 50, 51, 53	**1.29 (1.04–1.60)**	27.33	<0.001	74.4
Overall studies							
Processed meat	26	9917	14, 16, 17, 18, 19, 22, 23, 24, 25, 28, 29, 31, 32, 37, 39, 41, 42, 44, 45, 46, 48, 49, 50, 51, 53, 54	**1.45 (1.26–1.65)**	64.07	<0.001	61.0
Bacon	7	1641	15, 20, 23, 31, 34, 35, 45	**1.37 (1.17–1.61)**	4.13	0.659	0
Ham	5	1134	20, 23, 27, 31, 35	**1.44 (1.00–2.06)**	18.11	0.001	77.9
Sausage	9	3293	20, 21, 23, 27, 30, 33, 34, 35, 47	**1.33 (1.16–1.52)**	19.52	0.012	59.0
Subgroup analyses for processed meat							
High-quality studies (score≥7)	17	6932	14, 16, 17, 18, 19, 22, 23, 24, 25, 31, 32, 41, 44, 45, 46, 48, 50	**1.26 (1.10–1.46)**	33.59	0.006	52.4
Study design							
Cohort studies	9	3902	14, 16, 17, 18, 19, 22, 23, 24, 25	**1.18 (1.00–1.38)**	15.77	0.046	49.3
Case-control studies	18	6309	28, 29, 31, 31, 37, 39, 41, 42, 44, 45, 46, 48, 49, 50, 51, 53, 54	**1.64 (1.47–1.83)**	26.55	0.065	36.0
Population-based controls	8	2395	32, 37, 39, 41, 42, 45, 50, 54	**1.42 (1.19–1.70)**	8.29	0.308	15.6
Hospital-based controls	10	3914	28, 29, 31, 32, 44, 46, 48, 49, 51, 53	**1.79 (1.55–2.10)**	14.39	0.109	37.4
Sex							
Men	7	2021	14, 15, 16, 18, 25, 45, 46	**1.26 (1.09–1.46)**	9.58	0.144	37.3
Women	7	1517	16, 18, 23, 25, 44, 45,46	1.16 (0.99–1.36)	10.24	0.115	41.4
Histologic subtype							
Adenocarcinoma	11	4349	22, 24, 25, 31, 32, 37, 39, 42, 45, 46, 50	**1.42 (1.18–1.71)**	22.20	0.014	55.0
Anatomical subtype							
Cardia	4	968	22, 24, 25, 50	0.95 (0.76–1.19)	3.08	0.379	2.7
Non-cardia	4	1515	22, 24, 25, 50	**1.27 (1.07–1.52)**	5.16	0.160	41.9
Geographic Region							
Asia	7	1857	19, 28, 32, 42, 44, 48, 54	**1.58 (1.06–2.37)**	13.46	0.036	55.4
Europe	8	2482	17, 22, 23, 25, 29, 31, 41, 49	**1.50 (1.18–1.91)**	16.28	0.023	57.0
North America	8	4843	14, 16, 18, 24, 37, 45, 50, 51	**1.17 (1.06–1.29)**	13.42	0.063	47.8
Latin America	3	735	39, 46, 53	**1.94 (1.49–2.52)**	2.50	0.287	20.0
Outcome							
Incidence	24	8452	14, 16, 17, 22, 23, 24, 25, 28, 29, 31, 31, 37, 39, 41, 42, 44, 45, 46, 48, 49, 50, 51, 53, 54	**1.47 (1.27–1.69)**	55.44	<0.001	58.5
Adjustments							
Smoking, yes	15	7669	17, 18, 22, 24, 25, 32, 39, 42, 44, 45, 48, 49, 50, 51, 53	**1.36 (1.14–1.63)**	46.45	<0.001	69.9
Alcohol drinking, yes	9	4811	22, 23, 24, 25, 42, 48, 49, 50, 51	**1.51 (1.22–1.86)**	25.05	0.002	68.1
Total energy intake, yes	12	4573	17, 22, 23, 24, 25, 31, 39, 41, 45, 46, 50, 53	**1.38 (1.14–1.66)**	28.31	0.003	61.1
Family history, yes	6	3131	18, 41, 44, 45, 49, 53	1.25 (0.91–1.71)	14.15	0.015	64.7
BMI, yes	10	6193	18, 23, 24, 25, 41, 46, 49, 50, 51, 53	**1.41 (1.19–1.68)**	29.90	<0.001	69.9

a RR  =  relative risk (odds ratio); CI  =  confidence interval; BMI  =  body mass index.

### Processed meat and gastric cancer

**Figure 4:**
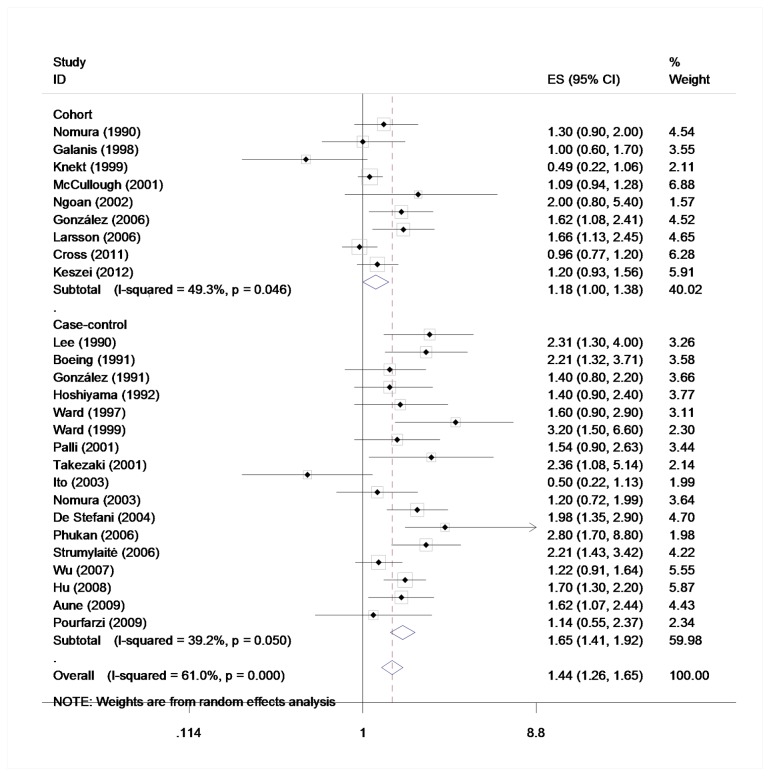
Estimates (95% CIs) of beef intake (highest versus lowest category) and gastric cancer risk. Squares indicate study-specific relative risks (size of the square reflects the study-specific statistical weight, i.e., the inverse of the variance); horizontal lines indicate 95% confidence intervals; diamond indicates summary relative risk estimate with its corresponding 95% confidence interval.

**Figure 5:**
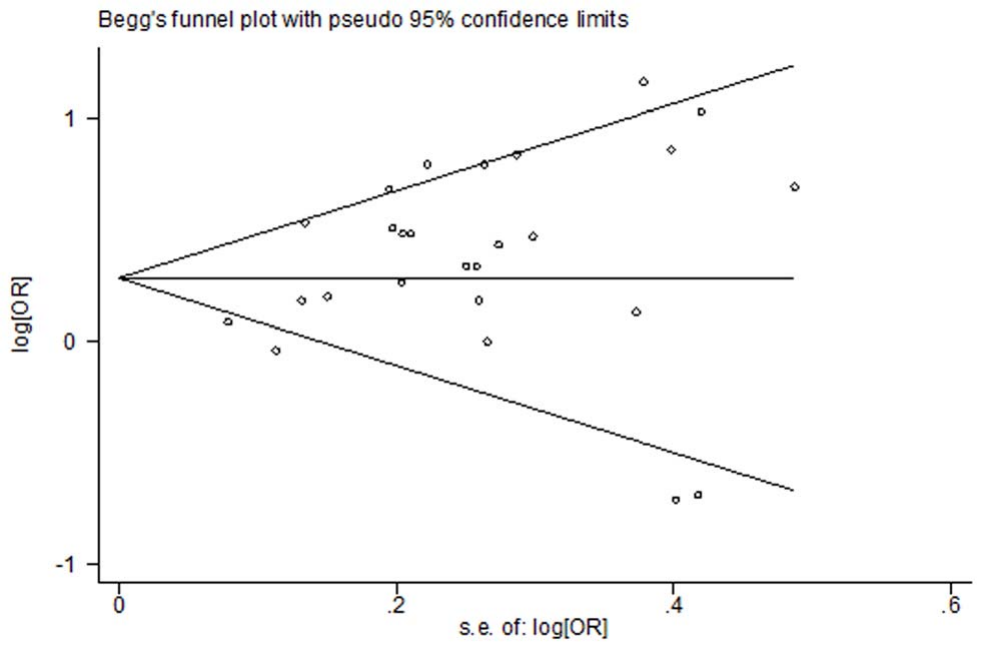
Beeg's test of studies for processed meat intake and gastric cancer risk.

Based on nine [14], [16]–[19], [22]–[25] cohort studies and seventeen [28], [29], [31], [32], [37], [39], [41], [42], [44]–[46], [48]–[51], [53], [54] case-controls in the highest versus lowest model, the meta-analysis of gastric cancer and processed meat yielded a summary RR of 1.45 (95% CI = 1.26–1.65). (Figure 4) Statistically significant heterogeneity was detected (*Q* = 64.07, *P*<0.001, *I^2^* = 61.0%), and publication bias was indicated from Egger's test (*P* = 0.037) but not Beeg's test (*P* = 0.467) (Figure 5). In the analysis of individual processed meat items, positive association was found between gastric cancer risk and bacon (RR = 1.37, 95% CI = 1.17–1.61), ham (RR = 1.44, 95% CI = 1.00–2.06), and sausage (RR = 1.33, 95% CI = 1.16–1.52). No heterogeneity was detected for bacon (*Q* = 4.13, *P* = 0.695, *I^2^* = 0%), while statistically heterogeneity was detected for ham (*Q* = 18.11, *P* = 0.001, *I^2^* = 77.9%) and sausage (*Q* = 19.52, *P* = 0.012, *I^2^* = 59.0%). No indication of publication bias of bacon (*P* = 0.512) and ham (*P* = 0.314) was observed form Egger's test and publication bias was found among sausage (*P* = 0.028).

The positive association was observed across cohort (RR = 1.18, 95% CI = 1.00–1.38), case-control (total: RR = 1.64, 95% CI = 1.47–1.83; population controls: RR = 1.42, 95% CI = 1.19–1.70; hospital controls: RR = 1.79, 95% CI = 1.55–2.01) and high-quality (RR = 1.26, 95% CI = 1.10–1.46) studies. A 26% increment of risk estimates was seen among men (RR = 1.26, 95% CI = 1.09–1.46), and no significant association among women (RR = 1.16, 95% CI = 0.99–1.36). The result of adenocarcinomas (RR = 1.42, 95% CI = 1.18–1.71) was consistent with the overall analysis. In the subgroup analysis by anatomical subtype, we found positive results of noncardia (RR = 1.27, 95% CI = 1.07–1.52), but null results of cardia (RR = 0.95, 95% CI = 0.76–1.19). When striated by geographic region, increment of risk estimates were found among Asian (RR = 1.58, 95% CI = 1.06–2.37), European (RR = 1.50, 95% CI = 1.18–1.91), North American (RR = 1.27, 95% CI = 1.06–1.52) and Latin American (RR = 1.94, 95% CI = 1.49–2.52) populations. When excluded the two [18], [19] studies of mortality, the results of incidence (RR = 1.47, 95% CI = 1.27–1.69) is consistent with the overall results. The positive association was statistically significant in studies those adjusted for smoking (RR = 1.36, 95% CI = 1.14–1.63), alcohol drinking (RR = 1.51, 95% CI = 1.22–1.86), total energy intake (RR = 1.38, 95% CI = 1.14–1.66), and BMI (RR = 1.41, 95% CI = 1.19–1.68), while not in that adjusted for family history (RR = 1.25, 95% CI = 0.91–1.71). (Table 1).

## Discussion

To our knowledge, there is the first meta-analysis to report an association between red meat intake and gastric cancer risk, which is also an updated meta-analysis to report the association between processed meat intake and gastric cancer risk since a previous study [67] published in 2006. Our findings indicated that red and processed meat is associated with a 45% increased gastric cancer risk separately when the highest reported intake was compared with the lowest. In the analysis of individual meat items, high beef, bacon, ham and sausage consumption are associated with increased gastric cancer risk, while no association was found among pork, indicating that meat type probably make a difference.

Suggested biologically mechanisms for the positive increased association between red and processed meat intake and gastric cancer include heme iron, which is much more abundant in red meat than white meat [68]. Heme iron contributes to endogenous formation of carcinogenic *N*-nitroso compounds (NOC), which have been linked to gastric cancer in epidemiological studies [69]. And oxidative stress and DNA damage caused by iron is thought to be an essential growth factor for *Helicobacter pylori* [70]. Another risk factor is the salt in cooking, processing and persevering meat. Excepting for introducing mutagens and carcinogens, experimental data suggest that high salt intake can damage the gastric mucosa and lead to inflammation [71]. Nitrate and nitrite compounds in processed meat also contribute to the formation of *N*-nitroso compounds [72]. Moreover, high temperature during cooking meat may produce heterocyclic amines and polycyclic aromatic hydrocarbons [73].

We showed that the magnitude of risk increment reported in high-quality studies was not as strong as that reported in the overall analysis (a 30% compared with 45% risk increment for red meat and a 26% compared with 45% risk increment for processed meat), which indicated that the association may have been enhanced by poor study methodologies. In subgroup analysis by study design, case-control studies, especially hospital-based case-control studies seems to reported much higher relative risks than cohort studies. The inconsistent findings may have been attributed to greater recall and selection biases in case-control studies because of their retrospective nature. And most non-high-quality studies are case-control ones, which further explain these results. When stratified by sex, increment relative risk was only observed among men for processed meat, probably because men consume more processed meat than women. When stratified by histological subtype, positive association was found a 28% and 42% increased relative risk of gastric adenocarcinomas and red and processed meat intake, which is consistent with the overall findings. It is interesting that increased relative risk was seen among red meat intake and cardic cancer, as well as processed meat intake and non-cardia cancer. Red and processed meat intake may have different impact on cardia and non-cardia cancers. When stratified by geographic region, 17%–94% increment was found among Asian, European, and North American and Latin American populations for processed meat intake and a 52% increment among European populations for red meat, probably indicating that ethnicity or regional lifestyle may have some effect. Based on the results of adjustments, the potentially important confounding factors of smoking, total energy intake, family history and BMI are excluded in the analysis of red meat and smoking, alcohol drinking, total energy intake and BMI are excluded in the analysis of processed meat. What has to be point out is that estimation of the four [23]–[25] cohort studies of red meat and gastric cancer risk (RR = 1.02, 95% CI = 0.90–1.17) is basically different with the conclusion drawn from case-control studies and the general analysis. This discrepancy can due to selection bias and information bias of retrospective case-control studies. However, considering the much smaller size of cohort studies than case-control ones, the results still needs further investigation. Compared with red meat, the increased association between processed meat and gastric cancer risk had stronger evidence, such as evidence from cohort studies and other subgroups. We assume that the increased association between red meat and gastric cancer risk still needs evidence from well-designed prospective cohort studies. Meanwhile, processed meat in the market is mainly made from red meat. In this means, we assume that processed meat or the processing method may play a greater role than red meat itself.

As mentioned previously, a study [67] was published in 2006 to investigate the possible relationship between gastric cancer and processed meat intake. The estimated summary relative risks of gastric cancer for the highest versus lowest intake of processed meat was 1.37 (95% CI = 1.17–1.61). In our study, the summary relative risk of processed meat was 8% higher (RR = 1.45, 95% CI = 1.26–1.65), which implicating that articles published after 2006 strengthened the positive association. There is no update of studies of bacon, ham, and sausage. Also, we carried out research on red meat in the method of meta-analysis originally and found statistically significant associations. The subgroup analyses in our study provided comprehensive results.

Strengths of our studies include a large size (2343450 participants and 5118 gastric cancer cases from cohort studies, and 11680 cases and 67544 controls from case-control studies). However, our meta-analysis still has several limitations. First, the association between red/processed meat consumption and stomach cancer risk is statistically significantly stronger in the case-control studies than in the cohort studies. Prospective cohort studies are less susceptible to bias due to information on exposures is collected before the diagnosis of the disease. Case-control studies, especially hospital-based ones may have concerned selection bias of controls. The overall association may have been overstated. Second, because of inability to fully adjust for various confounders, the increased risk of red/processed meat on gastric cancer could be attributed to other factors such as, alcohol drinking, family history, BMI, *et al*. The important risk factor *Helicobacter pylori* infection status was adjusted for one study [53]. Third, because of a board classification of red/processed meat in each component studies our findings were likely to be influenced by the misclassification of meat. The item “red meat” in some studies may include some processed red meat while some just contains fresh red meat. And some studies provide results of some specific kinds of red/processed meat. Fourth, the intake quantity in each study varies, including grams/day, times/week, grams/1000 kcal, quartiles, quintiles, *et al*. The highest and lowest intake varies across studies. The highest intake in one study may be similar to the median or lowest in another, which could cause bias to the overall results. And because of different methods used to assess and report red/processed meat intake across studies, we failed to evaluate a dose-response relation between red/processed intake and gastric cancer. Fifth, as many meta-analyses, publication bias and substantial heterogeneity exist in the component studies, which may due to study design, study populations, analytic strategies and other unknown factors. Thus, the summary results may be an overestimate of the relative risk of gastric cancer associated with red/processed consumption.

In conclusion, our analysis indicates that red and/or processed intake is associated with higher gastric cancer risk. Processed meat or the processing method itself may play a greater role in this contribution than red meat. However, the findings from our study need to be confirmed in future research in well-designed cohort or intervention studies. In addition, the underlying mechanisms call for further elucidation.

## Supporting Information

Figure S1 (DOC)

Table S1 (DOC)

Table S2 (DOC)

Table S3 (DOC)

Table S4 (DOC)
