# Supplementary material for: Red and Processed Meat Intake Is Associated with Higher Gastric Cancer Risk: A Meta-Analysis of Epidemiological Observational Studies
Source: PLoS One. 2013 Aug 14;8(8):e70955. doi: 10.1371/journal.pone.0070955 (PMC3743884; doi:10.1371/journal.pone.0070955)
Supplement: Figure S1 — (DOC) [file pone.0070955.s001.doc]

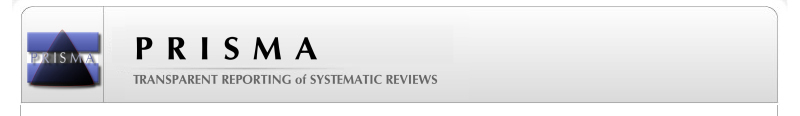
**PRISMA 2009 Flow Diagram**

**Screening**

**Included**

**Eligibility**

**Identification**

Records identified through database searching
(n =248)

Additional records identified through other sources
(n =12)

Records after duplicates removed
(n =146)

Records screened
(n =64 )

Records excluded
(n =82)

Full-text articles assessed for eligibility
(n =53)

Full-text articles excluded, with reasons
(n =11 )

Studies included in qualitative synthesis
(n =42 )

Studies included in quantitative synthesis (meta-analysis)
(n =42 )
