# Supplementary material for: Red and Processed Meat Intake Is Associated with Higher Gastric Cancer Risk: A Meta-Analysis of Epidemiological Observational Studies
Source: PLoS One. 2013 Aug 14;8(8):e70955. doi: 10.1371/journal.pone.0070955 (PMC3743884; doi:10.1371/journal.pone.0070955)
Supplement: Table S1 — (DOC) [file pone.0070955.s002.doc]

Supplemental Table 1 Characteristics of prospective cohort studies of red and processed meat and gastric cancer risk ^a^

| Author, year, region (reference) | Journal | No. of cases  (outcome) | Cohort size, cohort name and duration of follow-up | Type of cancer | Type of meat | Consumption categories | Adjusted RR (95%CI) | Adjusted variables | Quality score (0-10) |
| --- | --- | --- | --- | --- | --- | --- | --- | --- | --- |
| Nomura A, 1990, USA (14) | *Cancer Res* | 150 (incidence) | 7990 men (Japanese ancestry), Honolulu Heart Program, 20y | Gastric cancer | Processed meat (ham, bacon, and sausage)  (times/week) | <1  2-4  ≥5 | 1.0 (Referent)  1.0 (0.7-1.4)  1.3 (0.9-2.0) | Age | 8 |
| Kneller RW, 1991, USA (15) | *Cancer* | 75 (mortality) | 17 633 men, 20 y | Gastric cancer | Bacon, and side pork  (times/month) | <3  3-5  6-13  ≥14 | 1.0 (Referent)  1.7 (0.88-3.27)  2.0 (1.02-3.90)  1.4 (0.63-3.06) | Age and smoking | 6 |
| Galanis D J, 1998, USA (16] | *Int J Epdemiol* | 108 (incidence) | 11907 men and women (Hawaiian Japanese), 14.8y | Gastric cancer | Processed meat  (times/week) | 0  1-2  ≥3 | 1.0 (Referent)  0.9 (0.6-1.4)  1.0 (0.6-1.7) | Age, sex, education, and Japanese place of birth | 8 |
| Knekt P, 1999, Finland (17) | *Int J Cancer* | 68 (incidence) | 9989 men and women, 24y | Gastric cancer | Processed meat (cured meat)  Quartiles | Q1(low)  Q2  Q3  Q4(high) | 1.0 (Referent)  0.79 (0.41-1.54)  1.22 (0.67-2.23)  0.49 (0.22-1.06) | Age, sex, municipality, smoking, and energy intake | 9 |
| McCullough ML, 2001, USA (18) | *Cancer Epidemiol Biomarkers Prev* | 1349 (910 men, 439 women) (mortality) | 970045 (436654 men, 533391 women), Cancer Prevention Study (CPS) II, 14y | Gastric cancer | Processed meat  (times/week) | <1  1.0-4.5  ≥4.5  <1.5  1.5-3  ≥3 | Men  1.0 (Referent)  1.03 (0.86-1.23)  1.08 (0.87-1.33)  Women  1.0 (Referent)  0.99 (0.79-1.24)  1.11 (0.88-1.39) | Age, education, smoking, BMI, race, vitamin C and multivitamins use, aspirin use, and family history | 9 |
| Ngoan LT, 2002, Japan (19) | *Br J Cancer* | 116 (mortality) | 13250 men and women, 10.5y | Gastric cancer | Processed meat | <2-4/month  2-4/week  ≥1/day | 1.0 (Referent)  0.7 (0.3-1.3)  2.0 (0.8-5.4) | Age, sex, and intake of liver, oil, suimono, and picked foods | 8 |
| van der Brandt PA, 2003, Netherlands  (20) | *Cancer Cause Control* | 282 (incidence) | 3123 men and women, subcohort of The Netherlands Cohort Study, 6.3 y | Gastric cancer | Bacon  (grams/day)  Ham , boiled  (grams/day)  Sausage  (grams/day) | 0  >0  0  0.1-5  >5  0  0.1-3  >3.0 | 1.0 (Referent)  1.33 (1.03-1.71)  1.0 (Referent)  0.78 (0.58-1.05)  0.77 (0.56-1.07)  1.0 (Referent)  0.86 (0.63-1.17)  0.95 (0.67-1.35) | Age, sex, education, stomach disorders, family history, and smoking | 7 |
| Tokui N, 2005 Japan (21) | *J Epidemiol* | 859 (574 men, 285 women) (mortality) | 110 792 men and women, The Japanese Collaborative Cohort Study (JACC Study), 11y | Gastric cancer | Beef  Pork  Sausage | None  1-2/month  1-2/week  3-4/week  1+/day  None  1-2/month  1-2/week  3-4/week  1+/day  None  1-2/month  1-2/week  3-4/week  1+/day  None  1-2/month  1-2/week  3-4/week  1+/day  None  1-2/month  1-2/week  3-4/week  1+/day  None  1-2/month  1-2/week  3-4/week  1+/day | Men  1.0 (Referent)  0.79 (0.61-1.04)  0.73 (0.55-0.96)  1.00 (0.67-1.47)  0.90 (0.39-2.05)  Women  1.0 (Referent)  1.02 (0.69-1.51)  1.09 (0.74-1.62)  0.92 (0.50-1.69)  2.05 (0.74-5.68)  Men  1.0 (Referent)  0.96 (0.66-1.47)  1.10 (0.78-1.55)  1.10 (0.74-1.62)  1.05 (0.57-1.93)  Women  1.0 (Referent)  1.13 (0.69-1.85)  1.16 (0.74-1.82)  1.35 (0.81-2.26)  1.50 (0.65-3.48)  Men  1.0 (Referent)  0.93 (0.71-1.22)  1.00 (0.77-1.28)  1.24 (0.92-1.67)  1.36 (0.85-2.20)  Women  1.0 (Referent)  1.31 (0. 90-1.91)  1.13 (0.78-1.64)  1.18 (0.74-1.89)  1.82 (0.90-3.70) | Age | 5 |
| González CA, 2006, Europe (22) | *J Natl Cancer Inst* | 348 (incidence) | 521457 men and women, European Prospective Investigation Into Cancer and  Nutrition (EPIC) cohort, 6.5y | Gastric adenocarcinomas (cadia, non-cardia, intestinal, and diffuse) | Red meat  (grams/day)  Processed meat  (grams/day) | 0-26, 0-17 ^b^  26-52, 17-36  52-84, 36-61  84-1087, 61-584  Per 50 g/d  0-16, 0-9  16-34, 9-20  34-59, 20-37  59-730, 37-771  Per 50 g/d | 1.0 (Referent)  1.22 (0.87-1.71)  1.27 (0.89-1.82)  1.50 (1.02-2.22)  1.31 (0.89-1.94)  1.0 (Referent)  1.10 (0.76-1.58)  1.16 (0.79-1.69)  1.62 (1.08-2.41)  1.64 (1.07-2.51) | Sex, height, weight, education, alcohol use, smoking, physical activity, energy intake, fruits and vegetable intake, and other meats intake. | 9 |
| Larsson SC, 2006, Sweden (23) | *Int J Cancer* | 156 (incidence) | 61433 women, Swedish Mammography Cohort, 18y | Stomach cancer | Red meat (beef,  pork, lamb or veal)  (times/week)  Processed meat (Bacon, side pork, sausage, hot dogs, ham, or salami)  (times/week)  Bacon, and side pork  (times/week)  Ham, and salami  (times/week)  Sausage, and hot dogs  (times/week) | <2.0  2.0-3.4  ≥3.5  Per 10 g/d  <1.5  1.5-2.9  ≥3.0  Per 10 g/d  0  0.1-0.4  ≥0.5  <0.4  0.4-1.4  ≥1.5  <0.4  0.4-.09  ≥1.0 | 1.0 (Referent)  1.07 (0.73-1.57)  1.07 (0.69-1.66)  0.79 (0.60-1.05)  1.0 (Referent)  1.46 (0.95-2.25)  1.66 (1.13-2.45) ^c^  1.02 (0.96-1.08)  1.0 (Referent)  1.27 (0.88-1.85)  1.55 (1.00-2.41)  1.0 (Referent)  0.97 (0.65-1.51)  1.48 (0.99-2.22)  1.0 (Referent)  1.44 (0.89-2.35)  1.50 (0.93-2.41) | Age, education, BMI, energy intake, alcohol, fruits and vegetables intake | 10 |
| Cross AJ, 2011, USA (24) | *Am J Gastroenterol* | 955 (454 cardia, 501 non-cardia) (incidence) | 494979 men and women, The NIH-AARP Diet and Health study, 10y | Gastric adenocarcinomas (cardia and non-cardia) | Red meat  Quintile median (grams/1000kcal)  Processed meat  Quintile median (grams/1000kcal) | 10.0 ^d^  21.9  32.2  44.1  64.8  Per 10g/1000kcal  10.0  21.9  32.2  44.1  64.8  Per 10g/1000kcal  1.7  4.5  7.8  12.6  23.2  Per 10g/1000kcal  1.7  4.5  7.8  12.6  23.2  Per 10g/1000kcal | Cardia  1.0 (Referent)  1.29 (0.92-1.81)  1.12 (0.79-1.59)  1.13 (0.79-1.61)  1.04 (0.72-1.51)  1.00 (0.95-1.04)  Non-cardia  1.0(Referent)  0.81 (0.61-1.08)  0.72 (0.53-0.97)  0.83 (0.61-1.11)  0.77 (0.56-1.06)  0.99 (0.94-1.04)  Cardia  1.0 (Referent)  0.89 (0.64-1.24)  0.91 (0.66-1.26)  0.92 (0.67-1.28)  0.82 (0.59-1.14)  1.00 (0.92-1.09)  Non-cardia  1.0 (Referent)  0.87 (0.64-1.18)  1.10 (0.82-1.47)  1.04 (0.77-1.41)  1.09 (0.81-1.48)  1.02 (0.94-1.11) | Age, education, sex, BMI, ethnicity, smoking, alcohol drinking, physical activity daily intake of fruits , vegetables, saturated fat and calories | 10 |
| Keszei AP, 2012, Netherlands (25) | *Ann Oncol* | 652 (139 cardia and 329 non-cardia men, 24 cardia and 160 non-cardia women) (incidence) | 120852 (58279 men, 62573 women), The Netherlands Cohort Study on Diet and Cancer, 16.3y | Gastric adenocarcinomas (cardia and non-cardia) | Red meat (beef, pork, minced meat, liver, and other non-poultry meat)  Quintile/tertile median  (grams/day)  Processed meat (sausage, bacon, ham, cold cuts, croquettes, and frankfurters)  Quintile/tertile median  (grams/day) | 45.8  72.5  89.3  107.5  145.9  Per 50 g/d  45.8  72.5  89.3  107.5  145.9  Per 50 g/d  46.9  77.9  115.9  Per 50 g/d  46.9  77.9  115.9  Per 50 g/d  3.7  10.8  17.2  26.1  45.5  Per 50 g/d  3.7  10.8  17.2  26.1  45.5  Per 50 g/d  3.5  11.9  26.0  Per 50 g/d  3.5  11.9  26.0  Per 50 g/d | Men, cardia  1.0 (Referent)  0.90 (0.50-1.59)  1.16 (0.67-2.01)  1.01 (0.56-1.80)  1.00 (0.56-1.78)  0.98 (0.77-1.25)  Men, non-cardia  1.0 (Referent)  1.16 (0.79-1.72)  0.90 (0.60-1.37)  1.32 (0.9-1.94)  1.15 (0.77-1.71)  1.05 (0.91-1.22)  Women, cardia  1.0 (Referent)  0.61 (0.24-1.56)  0.45 (0.17-1.19)  0.77 (0.39-1.49)  Women, non-cardia  1.0 (Referent)  0.75 (0.50-1.12)  0.85 (0.57-1.26)  0.96 (0.75-1.23)  Men, cardia  1.0 (Referent)  1.51 (0.86-2.64)  0.89 (0.47-1.68)  1.26 (0.71-2.24)  1.49 (0.81-2.75)  1.15 (0.71-1.86)  Men, non-cardia  1.0 (Referent)  1.05 (0.71-1.56)  0.96 (0.64-1.44)  1.09 (0.64-1.44)  1.19 (0.78-1.79)  1.15 (0.83-1.59)  Women, cardia  1.0 (Referent)  1.19 (0.41-3.44)  1.12 (0.36-3.47)  0.70 (0.14-3.47)  Women, non-cardia  1.0 (Referent)  1.21 (0.81-1.81)  1.11 (0.73-1.70)  1.02 (0.54-1.93) | Age, smoking, energy intake, BMI, alcohol intake, vegetable intake, fruit intake, education and non-occupational physical activity | 10 |

a RR = relative risk (rate ratio or hazard ratio); CI = confidence interval; BMI = body mass index.

b Range of consumption among men and women, respectively.

c In a subanalysis of participants for whom data on smoking were available (including 52 stomach cancer patients), the multivariable RR after further adjustments for smoking was 1.65 (0.80-3.39) for ≥3 servings/week versus <1.5 servings/week.

d Median consumption among quintiles or tertiles.
