# Supplementary material for: Red and Processed Meat Intake Is Associated with Higher Gastric Cancer Risk: A Meta-Analysis of Epidemiological Observational Studies
Source: PLoS One. 2013 Aug 14;8(8):e70955. doi: 10.1371/journal.pone.0070955 (PMC3743884; doi:10.1371/journal.pone.0070955)
Supplement: Table S2 — (DOC) [file pone.0070955.s003.doc]

Supplemental Table 2 Characteristics of case-controls studies of red and processed meat and gastric cancer risk ^a^

| Author, year, region (reference) | Journal | No. of cases | No. and type of control subjects | Type of cancer | Type of meat | Consumption categories | Adjusted OR (95%CI) | Adjusted variables | Quality score  (0-10) | |
| --- | --- | --- | --- | --- | --- | --- | --- | --- | --- | --- |
| Risch HA, 1985,  Canada (26) | *Am J Epidemiol* | 246 (incidence) | 246 population-based  control subjects | Gastric cancer | Processed meat (smoked, salted, and pickled meat) | Per 100 g/d | 3.92 (1.76-8.75) | Age, sex, ethnicity, intakes of grains, chocolate, fibrous foods, egg, and public water supply | | 4 |
| La Vecchia C, 1987, Italy (27) | *Int J Cancer* | 206 (incidence) | 474 hospital-based  control subjects | Gastric cancer | Beef  Ham  Sausage | Low  Intermediate  High  Low  Intermediate  High  Low  Intermediate  High | 1.0 (Referent)  0.45 (0.29-0.63) b  0.86 (0.53-1.20) b  1.0 (Referent)  1.27 (0.9-1.8) ^b^  1.60 (1.1-2.3) ^b^  1.0 (Referent)  0.56 (0.4-0.9) ^b^  1.27 (0.9-1.7) ^b^ | Age, sex, education, residence and intakes of sugar, pasta/rice, polenta, whole grain bread/pasta, fruits, and vegetables | | 6 |
| Lee HH, 1990,  Taiwan (28) | *Anticancer Res* | 210 (incidence) | 810 hospital-based  control subjects | Gastric cancer | Processed meat (cured meat)  (meals/month) | <1  1  ≥2 | 1.0 (Referent)  2.04 (1.3-3.3) ^b^  2.31 (1.3-4.0) ^b^ | Age, sex, and hospital | | 4 |
| Boeing H, 1991,  Germany (29) | *Int J Cancer* | 143 (incidence) | 579 hospital-based  control subjects | Gastric cancer (cardia, corpus, and antrum/pylorus) | Processed meat  Tertiles | T1  T2  T3 | 1.0 (Referent)  1.37 (0.82-2.31)  2.21 (1.32-3.71) | Age, sex, hospital, and intakes of cheese, whole meal bread, raw vegetables, and citrus fruits | | 5 |
| Boeing H, 1991,  Poland (30) | *Cancer Cause Control* | 741 (incidence) | 741 population-based  control subjects | Gastric carcinoma (intestinal and diffuse type) | Sausage | Low  Intermediate  High | 1.0 (Referent)  1.20 (0.95-1.51)  1.55 (1.07-2.26) | Age, sex, education, occupation, and  residence | | 4 |
| González CA, 1991, Spain (31) | *Int J Cancer* | 354 (incidence) | 354 hospital-based  control subjects | Gastric adenocarcinomas | Processed meat (cured meat)  Bacon  2 categories  Ham, boiled  2 categories | <3  3-25  26-56  >56 | 1.0 (Referent)  1.1 (0.6-1.9)  1.5 (0.9-2.4)  1.4 (0.8-2.2)  1.0 (Referent)  1.1 (0.7-1.6)  1.0 (Referent)  1.6 (1.1-2.2) | Age, sex, and intakes of preserved fish, egg, nuts, fruits, vegetables, and energy | | 7 |
| Hoshiyama Y, 1992, Japan (32) | *Cancer Cause Control* | 294 (incidence) | 294 population-based  control subjects,  202 hospital-based  control subjects | Gastric adenocarcinomas | Processed meat (Smoked food/bacon/ham)  (times/week) | None  ≤1  ≥2  None  ≤1  ≥2 | Population controls  1.0 (Referent)  1.7 (1.1-2.8)  1.4 (0.9-2.4)  Hospital controls  1.0 (Referent)  1.7 (1.0-2.9)  1.9 (1.0-3.3) | Age, sex, administrative division, and smoking | | 7 |
| Sanchez-Diez, 1992, Spain (33) | *Eur J Epidemiol* | 109 (incidence) | 123 population-based  control subjects | Gastric adenocarcinomas | Sausage, smoked | None  Daily | 1.0 (Referent)  3.55 (1.59-7.94) | Age, sex, and residence | | 4 |
| Hansson LE, 1993, Sweden (34) | *Int J Cancer* | 338 (incidence) | 669 population-based  control subjects | Gastric cancer | Bacon  (times/month)  Sausage  (times/month) | <1  1-3  4-7  >7  ≤3  4-7  >7 | 1.0 (Referent)  1.09 (0.72-1.65)  1.01 (0.67-1.52)  1.42 (0.90-2.23)  1.0 (Referent)  1.07 (0.75-1.53)  0.91 (0.63-1.33) | Age, sex, and socioeconomic status | | 7 |
| Nazario CM, 1993,  Puerto Rico (35) | *Int J Epidemiol* | 136 (incidence) | 151 population-based  control subjects | Gastric cancer | Beef, jerked  Pork (pig feet)  Bacon  Ham  Sausage | Ever versus never  Ever versus never  Ever versus never  Ever versus never  Ever versus never | 1.0 (Referent)  1.4 (0.9-2.2)  1.0 (Referent)  2.2 (1.3-3.8)  1.0 (Referent)  2.1 (1.2-3.5)  1.0 (Referent)  1.8 (1.1-2.8)  1.0 (Referent)  2.3 (1.4-3.7) | Sex | | 5 |
| Muñoz SE, 1997, Italy (36) | *Cancer Epidemiol Biomarkers Prev* | 88 (incidence) | 103 hospital-based subjects | Gastric cancer | Red meat  (times/week) | ≤2  3-4  ≥5 | 1.0 (Referent)  1.62 (0.81-3.26)  3.38(1.42-8.04) | Sex, age, area of residence, and education | | 5 |
| Ward MH, 1997, USA (37) | *Int J Cancer* | 176 (incidence) | 449 population-based subjects | Gastric adenocarcinomas (cardia and distal) | Red meat (beef, pork, liver, processed red meats)  (times/week)  Beef (steaks/roasts, hamburgers)  (times/week)  Processed meat (bacon, sausage, ham, cured meats, sandwich meats)  (times/week) | <8  8-12  13-18  >19  <3  3-4  5  6+  <4  4-5  6-8  >8 | 1.0 (Referent)  1.4 (0.7-2.9)  2.1 (1.1-4.2)  2.4(1.3-4.8)  1.0 (Referent)  1.5 (0.9-2.6)  1.8 (0.9-3.7)  1.6 (0.9-3.0)  1.0 (Referent)  1.4 (0.7-2.9)  1.3 (0.7-2.4)  1.6 (0.9-2.9) | Age, sex | | 5 |
| Ji BT, 1998, China (38) | *Int J Cancer* | 1124 (770 men, 353 women) (incidence) | 1451 (819 men, 632 women) population-based subjects | Gastric cancer | Red meat (pork chops, pork spareribs, pig feet, fresh pork, beef, and mutton)  (times/month) | ≤8.5  8.6-16.1  16.2-30.6  ≥30.7  ≤8.5  8.6-16.1  16.2-30.6  ≥30.7 | Men  1.0 (Referent)  0.9 (0.7-1.2)  1.1 (0.8-1.4)  0.9 (0.6-1,2)  Women  1.0 (Referent)  0.9 (0.6-1.3)  0.9 (0.6-1.3)  0.8 (0.6-1,2) | Age, income, education, smoking (males only), and alcohol drinking (males only) | | 5 |
| Ward MH, 1999, Mexico (39) | *Am J Epidemiol* | 220 (incidence) | 752 population-based  control subjects | Gastric adenocarcinomas | Processed meat  (times/week) | <1  1-2  3-5  ≥6 | 1.0 (Referent)  2.0 (1.0-3.6)  2.8 (1.4-5.7)  3.2 (1.5-6.6) | Age, sex, total calories, chill pepper consumption, added salt, history of peptic ulcer, smoking, and socioeconomic status | | 6 |
| Tavani A, 2000, Italy (40) | *Int J Cancer* | 745 (incidence) | 7990 hospital-based subjects | Gastric cancer | Red meat (excluding canned and preserved meat)  (portions^c^/week) | ≤3  3-≤6  >6  Per 1 portion/day | 1.0 (Referent)  1.1 (0.8-1.3)  1.6(1.3-2.0)  1.7 (1.3-2.2) | Age, year of recruitment, sex, education, smoking habits and alcohol, fat, fruit and vegetable intakes | | 6 |
| Palli D, 2001, Italy (41) | *Cancer Res* | 382 (incidence) | 561 population-based  control subjects | Gastric cancer with microsatellite instability | Red meat (beef/pork/  lamb/game)  Tertiles  Processed meat (cured & canned meats)  Tertiles | T1  T2  T3  T1  T2  T3  T1  T2  T3  T1  T2  T3 | MSI+  1.0 (Referent)  1.7 (0.6-4.6)  4.3 (1.8-10.8)  MSI–  1.0 (Referent)  0.9 (0.4-1.7)  2.1 (1.2-3.7)  MSI+  1.0 (Referent)  1.0 (0.5-2.4)  1.0 (0.4-2.6)  MSI–  1.0 (Referent)  1.2 (0.6-2.3)  1.9 (1.0-3.7) | Age, sex, social class, family history, residence, BMI, total energy, and consumption tertiles of each food of interest | | 7 |
| Takezaki T, 2001,  China (42) | *Jpn J Cancer Res* | 187 (incidence) | 333 population-based  control subjects | Gastric cancer | Processed meat (salted meat)  (times/month) | <1  1-3  ≥4 | 1.0 (Referent)  3.82 (2.24-6.50)  2.36 (1.08-5.14) | Age, sex, smoking, and drinking habits | | 6 |
| Kim HJ, 2002, South Korea (43) | *Int J Cancer* | 136 (incidence) | 136 hospital-based subjects | Gastric cancer | Red meat (grilled beef and pork over charcoal)  Quartiles  Beef  Quartiles  Pork  Quartiles | Q1  Q2-Q3  Q4  Q1  Q2-Q3  Q4  Q1  Q2-Q3  Q4 | 1.0 (Referent)  1.28 (0.72-2.30)  1.58 (0.80-3.10)  1.0 (Referent)  0.78 (0.42-1.44)  1.67 (0.86-3.27)  1.0 (Referent)  0.80 (0.43-1.50)  0.94 (0.45-1.97) | Sex, age, socioeconomic status, family history, and refrigerator use | | 7 |
| Ito LS, 2003, Japan (44) | *Ann Epidemiol* | 508 (women only) (incidence) | 36 490 hospital-based  control subjects (women only) | Gastric cancer (differentiated and non-differentiated) | Beef  (times/week)  Pork  (times/week)  Processed meat  (times/week) | <1  1-2  3-4  ≥5  <1  1-2  3-4  ≥5  <1  1-2  3-4  ≥5 | 1.0 (Referent)  0.92 (0.76-1.11)  0.88 (0.63-1.24)  0.97 (0.39-2.39)  1.0 (Referent)  0.93 (0.76-1.13)  1.31 (1.01-1.72)  0.69 (0.28-1.68)  1.0 (Referent)  1.02 (0.83-1.26)  1.14 (0.83-1.57)  0.50 (0.22-1.13) | Age, year, season at first hospital visit,  family history, and smoking | | 7 |
| Nomura AM, 2003, USA (45) | *Cancer Cause Control* | 300 (186 men, 114 women) (incidence) | 446 (282 men, 164 women) population-based  control subjects | Gastric distal adenocarcinomas (intestinal, diffuse, and the mixed-other types) | Processed meat  (grams/day)  Bacon  (grams/day) | <9.2  9.2 – 27.2  >27.2  <6.1  6.1 – 14.6  >14.6  <0.1  0.1-1.5  >1.5  <0.1  0.1-0.7  >0.7 | Men  1.0 (Referent)  1.8 (1.0-3.3)  1.7 (0.9-3.3)  Women  1.0 (Referent)  0.6 (0.3-1.3)  0.7 (0.3-1.5)  Men  1.0 (Referent)  1.3 (0.7-2.2)  1.3 (0.7-2.4)  Women  1.0 (Referent)  0.6 (0.3-1.3)  1.1 (0.5-2.3) | Age, ethnicity, education, smoking,  history of gastric ulcer, NSAID use,  family history, intakes of other foods, and total calories | | 7 |
| De Stefani E, 2004, Uruguay (46) | *Gastric cancer* | 240 (incidence) | 960 hospital-based subjects | Gastric adenocarcinomas (cardia, fundus, body, lesser curvature, greater curvature, and antrum and pylorus) | Red meat (beef and lamb)  Tertiles  Processed meat (salted meat)  Tertiles | T1  T2  T3  T1  T2  T3 | 1.0 (Referent)  1.05 (0.70-1.56)  1.10(0.71-1.71)  1.0 (Referent)  1.32 (0.89-1.96)  1.98 (1.35-2.90) | Age, sex, residence, urban/rural status, education, BMI, and total energy intake | | 8 |
| Lissowska J, 2004, Poland (47) | *Nutr Cancer* | 274 (incidence) | 463 population-based  control subjects | Gastric cancer | Red meat (pork, beef, liver, and processed red meats)  (times/week)  Sausage and hot dogs  (times/week) | <8.0  8.0-11.1  11.2-14.5  >14.5  <2.1  2.1-3.4  3.5-4.9  >4.9 | 1.0 (Referent)  1.24 (0.79-1.95)  1.19 (0.73-1.92)  1.51 (0.90-2.51)  1.0 (Referent)  1.13 (0.74-1.71)  0.75 (0.48-1.17)  1.23 (0.79-1.93) | Age, sex, education, smoking, and calories from food. | | 8 |
| Phukan RK, 2006, India (48) | *J Gastroenterol* | 329 (incidence) | 658 hospital-based subjects | Gastric cancer | Beef  Pork  Processed meat (smoked dried salted meat) | Never  Occasional  Once a week  Twice or more a week  Never  Occasional  Once a week  Twice or more a week  Never  Occasional  Once a week  Twice or more a week | 1.0 (Referent)  0.23 (0.04-6.10)  0.46 (0.06-5.30)  0.89(0.03-9.40)  1.0 (Referent)  1.07 (0.06-6.27)  1.26 (0.02-9.11)  1.98(0.01-6.42)  1.0 (Referent)  1.6 (0.04-9.20)  2.1 (1.2-8.4)  2.8 (1.7-8.8) | Education, tobacco use, alcohol drinking, and each  dietary variable for another | | 7 |
| Strumylaitė L, 2006, Lithuania (49) | *Medicina (Kaunas)* | 379 (incidence) | 1139 hospital-based subjects | Gastric cancer | Processed meat (salted meat) | Almost do not use  1-3 times/month  ≥1-2 times/week | 1.0 (Referent)  1.85 (1.12-3.04)  2.21 (1.43-3.42) | Smoking, alcohol use, family history, BMI, education, residence, diet, and physical activity | | 5 |
| Wu AH, 2007, USA (50) | *Cancer Causes Control* | 623 (incidence) | 1308 population-based controls | Gastric adenocarcinoma (cardia and distal cardia) | Red meat  Quartiles  Processed meat  Quartiles | Q1  Q2  Q3  Q4  Q1  Q2  Q3  Q4  Q1  Q2  Q3  Q4  Q1  Q2  Q3  Q4 | Gastric cardia  1.0 (Referent)  1.20 (0.8-1.9)  1.70 (1.1-2.6)  1.56 (0.97-2.5)  Distal gastric  1.0 (Referent)  1.37 (0.9-2.0)  1.16 (0.8-1.7)  1.57 (1.0-2.4)  Gastric cardia  1.0 (Referent)  0.84 (0.6-1.3)  0.76 (0.5-1.2)  0.89 (0.6-1.4)  Distal gastric  1.0 (Referent)  1.54 (1.1-2.2)  1.22 (0.8-1.8)  1.65 (1.1-2.5) | Age, sex, race, birthplace, education, smoking, alcohol, BMI, reflux, use of vitamins and total calories | | 10 |
| Hu JF, 2008, Canada (51) | *Nutr Cancer* | 1182 (incidence) | 5039  population-based subjects | Gastric cancer | Red meat  (times/week)  Processed meat  (times/week) | ≤2  2.1-3.94  3.95-5  ≥5.1  ≤0,94  0.95-2.41  2.42-5.41  ≥5.42 | 1.0 (Referent)  1.1 (0.9-1.3)  1.1 (0.9-1.4)  1.2 (1.0-1.5)  1.0 (Referent)  1.2 (1.0-1.6)  1.3 (1.0-1.7)  1.7 (1.3-2.2) | Age, province, education, BMI, sex, alcohol use, smoking, fruit and vegetable intake | | 6 |
| Navarro Silvera SA, 2008, USA (52) | *Int J Cancer* | 607 (incidence) | 687  population-based subjects | Gastric adenocarcinoma (Cardia and non-cardia) | Red meat (beef, pork, veal, lamb, and liver) | Per one serving/day | Cardia  1.39 (0.80-2.42)  Non-cardia  1.37 (0.83-2.25) | Age, site, sex, race, region, proxy status, income, education, BMI, smoking, alcohol, and energy intake | | 7 |
| Aune D, 2009, Uruguay (53) | *Asian Pacific J Cancer Prev* | 275 (incidence) | 2032 hospital-based subjects | Gastric cancer | Red meat (fresh beef and lamb)  (grams/day)  Beef  (grams/day)  Processed meat  (grams/day) | 0-<150  150-<250  250-600  0-<150, 0-<90 ^d^  150-<250, 90-<150  250-524.8, 150-524.8  0-10  >10-40  >40-25.8 | 1.0 (Referent)  1.36 (0.98-1.89)  2.19 (1.31-3.65)  1.0 (Referent)  1.21 (0.88-1.65)  1.70 (1.04-2.83)  1.0 (Referent)  1.29 (0.89-1.86)  1.62 (1.07-2.44) | Age, sex, residence, education, income, interviewer, smoking, BMI, energy intake, variety of foods intake, and total energy | | 5 |
| Pourfarzi E, 2009, Iran (54) | *Int J Cancer* | 213 (incidence) | 390  population-based subjects | Gastric cancer (cardia and non-cardia) | Red meat  Processed meat | ≤2 times/week  3-4/week  >once/day  Never  ≥once/month | 1.0 (Referent)  2.20 (1.26-3.85)  3.40 (1.79-6.46)  1.0 (Referent)  1.14 (0.55-2.37) | Age, sex, education, family history, variety of foods intake and *H. pylori* | | 6 |
| Gao Y, 2011, China (55) | *Cancer Epidemiol* | 915 (incidence) | 1514 hospital-based controls | gastric adenocarcinomas (cardia and noncardia) | Red meat | Monthly/seldom/never  Weekly  >Weekly  Monthly/seldom/never  Weekly  >Weekly | cardia  1.0 (Referent)  1.21(0.95-1.55)  1.54(1.15-2.07)  noncardia  1.0 (Referent)  1.62(1.18-2.24)  1.77(1.21-2.58) | Age, gender, geographic region | | 5 |

a RR = relative risk (odds ratio); CI = confidence interval; BMI = body mass index; MSI=microsatellite instability; NSAID = nonsteroidal anti-inflammatory drug.

b Confidence intervals were calculated using the distribution of case patients and control subjects for each exposure category.

c an average Italian portion is 100 to 150g.

d Range of consumption among men and women, respectively.
