# Supplementary material for: Red and Processed Meat Intake Is Associated with Higher Gastric Cancer Risk: A Meta-Analysis of Epidemiological Observational Studies
Source: PLoS One. 2013 Aug 14;8(8):e70955. doi: 10.1371/journal.pone.0070955 (PMC3743884; doi:10.1371/journal.pone.0070955)
Supplement: Table S3 — (DOC) [file pone.0070955.s004.doc]

Supplemental Table 3 Methodological quality of cohort studies included in the meta-analysis ^a^

| First author, year | Representativeness of the exposed cohort | Selection of the unexposed cohort | Ascertainment of exposure | Outcome of interest not present at start of study | Control for important factor or additional factors ^b^ | Outcome assessment | Follow-up long enough for outcomes to occur ^c^ | Adequacy of follow-up of cohorts ^d^ | Data analysis that used an energy-adjusted residual or nutrient-density model | Total quality scores |
| --- | --- | --- | --- | --- | --- | --- | --- | --- | --- | --- |
| Nomura A, 1990 | ☆ | ☆ | ☆ | ☆ | ☆☆ | ☆ | ☆ | — | — | 8 |
| Kneller RW, 1991 | — | ☆ | ☆ | ☆ | ☆ | ☆ | ☆ | — | — | 6 |
| Galanis D J, 1998 | ☆ | ☆ | ☆ | ☆ | ☆ | ☆ | ☆ | ☆ | — | 8 |
| Knekt P, 1999 | ☆ | ☆ | — | ☆ | ☆☆ | ☆ | ☆ | ☆ | ☆ | 9 |
| McCullough ML, 2001 | ☆ | ☆ | ☆ | ☆ | ☆☆ | ☆ | ☆ | ☆ | — | 9 |
| Ngoan LT, 2002 | ☆ | ☆ | — | ☆ | ☆☆ | ☆ | ☆ | ☆ | — | 8 |
| van der Brandt PA, 2003, | ☆ | ☆ | ☆ | ☆ | ☆☆ | ☆ | — | — | — | 7 |
| Tokui N, 2005 | ☆ | ☆ | — | ☆ | — | — | ☆ | ☆ | — | 5 |
| González CA, 2006 | ☆ | ☆ | — | ☆ | ☆☆ | ☆ | ☆ | ☆ | ☆ | 9 |
| Larsson SC, 2006 | ☆ | ☆ | ☆ | ☆ | ☆☆ | ☆ | ☆ | ☆ | ☆ | 10 |
| Cross AJ, 2011 | ☆ | ☆ | ☆ | ☆ | ☆☆ | ☆ | ☆ | ☆ | ☆ | 10 |
| Keszei AP, 2012 | ☆ | ☆ | ☆ | ☆ | ☆☆ | ☆ | ☆ | ☆ | ☆ | 10 |

a A study could be awarded a maximum of one star for each item except for the item Control for important factor or additional factor.

b A maximum of 2 stars could be awarded for this item. Studies that controlled for smoking and alcohol received one star, whereas studies that controlled for other important confounders such as family history or fresh vegetables and fruit intake received an additional star.

c A cohort study with a follow-up time >8 y was assigned one star.

d A cohort study with a follow-up rate >75% was assigned one star.
