# Supplementary material for: Red and Processed Meat Intake Is Associated with Higher Gastric Cancer Risk: A Meta-Analysis of Epidemiological Observational Studies
Source: PLoS One. 2013 Aug 14;8(8):e70955. doi: 10.1371/journal.pone.0070955 (PMC3743884; doi:10.1371/journal.pone.0070955)
Supplement: Table S4 — (DOC) [file pone.0070955.s005.doc]

Supplemental Table 4 Methodological quality of case-control studies included in the meta-analysis ^a^

| First author, year [reference] | Adequate definition of cases | Representativeness of cases | Selection of control subjects | Definition of control subjects | Control for important factor or additional factors ^b^ | Exposure assessment | Same method of ascertainment for all subjects | Nonresponse rate ^c^ | Data analysis that used an energy-adjusted residual or nutrient-density model | Total quality scores |
| --- | --- | --- | --- | --- | --- | --- | --- | --- | --- | --- |
| Risch HA, 1985 | ☆ | ☆ | ☆ | — | — | — | ☆ | — | — | 4 |
| La Vecchia C, 1987 | ☆ | ☆ | — | ☆ | ☆ | — | ☆ | — | ☆ | 6 |
| Lee HH, 1990 | ☆ | ☆ | — | — | ☆ | — | ☆ | — | — | 4 |
| Boeing H, 1991(Germany) | — | ☆ | — | ☆ | ☆ | — | ☆ | — | ☆ | 5 |
| González CA, 1991 | ☆ | ☆ | — | ☆ | ☆ | ☆ | ☆ | — | ☆ | 7 |
| Boeing H, 1991 (Poland) | ☆ | ☆ | — | ☆ | — | — | ☆ | — | — | 4 |
| Sanchez-Diez, 1992 | ☆ | ☆ | ☆ | — | — | — | ☆ | — | — | 4 |
| Hoshiyama Y, 1992 | ☆ | ☆ | ☆ | ☆ | ☆ | ☆ | ☆ | — | — | 7 |
| Hansson LE, 1993 | ☆ | ☆ | ☆ | — | ☆☆ | ☆ | ☆ | — | — | 7 |
| Nazario CM, 1993 | ☆ | ☆ | ☆ | — | — | ☆ | ☆ | — | — | 5 |
| Muñoz SE, 1997 | ☆ | ☆ | — | ☆ | ☆ | — | ☆ | — | — | 5 |
| Ward MH, 1997 | ☆ | ☆ | ☆ | ☆ | — | — | ☆ | — | — | 5 |
| Ji BT, 1998 | — | ☆ | ☆ | — | ☆☆ | — | ☆ | — | — | 5 |
| Ward MH, 1999 | ☆ | ☆ | ☆ | — | ☆ | — | ☆ | — | ☆ | 6 |
| Tavani A, 2000 | ☆ | ☆ | — | ☆ | ☆☆ | — | ☆ | — | — | 6 |
| Takezaki T, 2001 | ☆ | ☆ | ☆ | — | ☆☆ | — | ☆ | — | — | 6 |
| Palli D, 2001 | ☆ | ☆ | ☆ | — | ☆☆ | — | ☆ | — | ☆ | 7 |
| Kim HJ, 2002 | ☆ | ☆ | — | ☆ | ☆☆ | ☆ | ☆ | — | — | 7 |
| Ito LS, 2003 | ☆ | ☆ | — | ☆ | ☆☆ | ☆ | ☆ | — | — | 7 |
| Nomura AM, 2003 | ☆ | ☆ | ☆ | — | ☆ | ☆ | ☆ | — | ☆ | 7 |
| Lissowska J, 2004 | ☆ | ☆ | ☆ | — | ☆☆ | ☆ | ☆ | — | ☆ | 8 |
| De Stefani E, 2004 | ☆ | ☆ | — | ☆ | ☆☆ | ☆ | ☆ | — | ☆ | 8 |
| Phukan RK, 2006 | ☆ | ☆ | — | ☆ | ☆ | ☆ | ☆ | — | ☆ | 7 |
| Strumylaitė L, 2006 | ☆ | ☆ | — | — | ☆ | — | ☆ | — | ☆ | 5 |
| Wu AH, 2007, USA | ☆ | ☆ | ☆ | ☆ | ☆☆ | ☆ | ☆ | ☆ | ☆ | 10 |
| Navarro Silvera SA, 2008 | ☆ | ☆ | ☆ | — | ☆☆ | — | ☆ | — | ☆ | 7 |
| Hu JF, 2008 | ☆ | ☆ | ☆ | — | ☆ | — | ☆ | — | ☆ | 6 |
| Aune D, 2009 | ☆ | ☆ | — | ☆ | ☆ | — | ☆ | — | — | 5 |
| Pourfarzi E, 2009 | ☆ | ☆ | ☆ | ☆ | ☆ | — | ☆ | — | — | 6 |
| Gao Y, 2011, China | ☆ | ☆ | — | ☆ | — | — | ☆ | ☆ | — | 5 |

a A study could be awarded a maximum of one star for each item except for the item Control for important factor or additional factor.

b A maximum of 2 stars could be awarded for this item. Studies that controlled for smoking and alcohol received one star, whereas studies that controlled for other important confounders such as family history or fresh vegetables and fruit intake received an additional star.

c One star was assigned if there was no significant difference in the response rate between control subjects and cases by using the chi-square test (*P*>0.05)
